# Nonlinear association of 1,5-anhydroglucitol with the prevalence and severity of coronary artery disease in chinese patients undergoing coronary angiography

**DOI:** 10.3389/fendo.2022.978520

**Published:** 2022-09-05

**Authors:** Ruiyue Yang, Wenduo Zhang, Xinyue Wang, Siming Wang, Qi Zhou, Hongxia Li, Hongna Mu, Xue Yu, Fusui Ji, Jun Dong, Wenxiang Chen

**Affiliations:** ^1^ The Key Laboratory of Geriatrics, Beijing Institute of Geriatrics, Institute of Geriatric Medicine, Chinese Academy of Medical Sciences, Beijing Hospital/National Center of Gerontology of National Health Commission, Beijing, China; ^2^ Department of Cardiology, Beijing Hospital, National Center of Gerontology; Institute of Geriatric Medicine, Chinese Academy of Medical Sciences, Beijing, China; ^3^ National Center for Clinical Laboratories, Institute of Geriatric Medicine, Chinese Academy of Medical Sciences, Beijing Hospital/National Center of Gerontology, Beijing, China

**Keywords:** 1,5-anhydroglucitol, hemoglobin a1c, diabetes, coronary artery diseases, genuine score

## Abstract

**Background:**

Postprandial hyperglycemia plays an important role in the pathogenesis of coronary artery disease (CAD). The aim of this study is to determine the associations of 1,5-Anhydroglucitol (1,5-AG), which reflects circulating glucose fluctuations, with the prevalence of CAD and CAD severity in coronary angiography defined Chinese patients.

**Methods:**

2970 Chinese patients undergoing coronary angiography were enrolled. Baseline demographics and medical history data was recorded. Serum 1,5-AG levels and biochemical parameters were measured. Baseline characteristics were compared across 1,5-AG categories in diabetes (DM) and non-DM groups. Logistic regression analysis was performed to evaluate the associations of 1,5-AG with the prevalence and severity of CAD.

**Results:**

Lower 1,5-AG was significantly associated with higher Gensini scores in both DM and non-DM groups. Logistic regression analysis demonstrated that the associations of low 1,5-AG with the prevalence of CAD, elevated Gensini score and severe CAD robustly dose-response increased from undiagnosed DM with 1,5-AG ≥ 14µg/mL to DM with 1,5-AG < 14µg/mL even after adjusting for fasting blood glucose (FBG) or Hemoglobin A1c (HbA_1c_). The associations were more significant in persons with DM. Significant modification effect of DM on the relationship of 1,5-AG with elevated Gensini score was found. In addition, nonlinear relationship and threshold effects of 1,5-AG with CAD and severity were observed.

**Conclusion:**

Low 1,5-AG is significantly and independently associated with CAD and CAD severity in Chinese patients undergoing coronary angiography. Measurement of 1,5-AG is useful to differentiate subjects with extensive glucose fluctuations and high CAD risks, especially in DM patients.

**Clinical Trial Registration:**

ClinicalTrials.gov, identifier NCT03072797.

## Introduction

Atherosclerotic coronary artery disease (CAD) is among the leading cause of mortality and morbidity and puts an enormous economic burden worldwide (1). There are multiple risk factors for CAD such as smoking, obesity, hypertension, hyperglycemia and dyslipidemia (2). Early identification and intervention of the risk factors are effective measures to the prevention and treatment of CAD. Type 2 diabetes mellitus (DM) is considered to be an important risk factor in the development of CAD (3). Therefore, intensive blood glucose control is critical to reduce the mortality and morbidity of CAD in DM patients. Hemoglobin A1c (HbA1c), which reflects glycemic exposure over the past 2–3 months, is the standard measure used for diagnosis of diabetes as well as the clinical monitoring of glucose control (4). However, in patients with established DM, intensive intervention for glycemic control guided by HbA1c values did not improve the risk of macro-vascular complications and survival prognosis (5, 6). Previous studies have shown that postprandial hyperglycemia and glycemic variability are risk factors, independent of average glycemic level, for cardiovascular complications in people with DM (7, 8). A growing body of literature suggests that 1,5-Anhydroglucitol (1,5-AG) may provide a useful complement to HbA1c measurements (9), especially when short-term glycemic variability may not be reflected in traditional glycemia markers.

1,5-AG is a dietary monosaccharide, a naturally occurring 1-deoxy form of glucose, that is typically present at high but stable concentrations in the blood in normal glycemic status (10). Serum concentrations of 1,5-AG is adjusted by urinary excretion in the kidneys. Most 1,5-AG, which is filtered in glomerulus is reabsorbed at a specific fructose-mannose active transporter in the renal tubule, with a small amount, corresponding to dietary intake, excreted in the urine (11). The reabsorption is competitively inhibited by glucose. Therefore, the serum 1,5-AG level rapidly decreases when serum glucose level exceeds the threshold of urine glucose excretion (160-180 mg/dL), even when very short-lasting episodes appear. Previous studies have demonstrated that 1,5-AG level is negatively correlated with HbA1c and fasting blood glucose (FBG) and is a useful marker to identify well controlled, exclusively based on HbA1c levels DM patients with transient hyperglycaemia (12, 13).

In community-based studies, low 1,5-AG level is associated with the prevalence of CAD and also has predictive value for CAD events and mortality, in both DM and non-DM populations (9, 14). However, it is unclear if 1,5-AG is associated with CAD and CAD severity in high risk subjects and if 1,5-AG adds prognostic value to HbA1c, especially in Chinese populations. CAD severity can be evaluated by the Gensini score system which reflect the complexity and extent of CAD based on the artery morphology, coronary anatomy, and severity of stenosis in lesions  (15, 16). Therefore, the present study was designed to determine the independent associations of 1,5-AG with the prevalence of CAD and CAD severity evaluated by Gensini score in Chinese patients underwent coronary angiography.

## Materials and methods

### Study population

From Mar, 2017 to 2020, 2970 consecutive patients undergoing coronary angiography in Beijing Hospital were enrolled in this study, in which 2945 subjects were measured serum 1,5-AG concentrations. The exclusion criteria were: (1) patients who had severe congenital heart disease, severe cardiac insufficiency, primary pulmonary hypertension, hepatic and renal dysfunction; (2) patients who were receiving chemotherapy or radiotherapy, pregnant or nursing; (3) patients who were alcohol or drug abuser, or with mental illness under treatment. Baseline demographics and medical history information, including height, weight, blood pressure, lifestyle, and history of DM, hypertension (HTN), stroke, and premature CAD were surveyed by trained doctors during hospitalization. Patients with HTN, DM and dyslipidemia include those who have already been diagnosed with these diseases at enrolment and those newly diagnosed during hospitalization according to current guidelines. Before coronary angiography, fasting blood samples were taken into common vacutainer tubes and serum was separated by centrifugation. Serum samples were aliquoted into 2mL vials and stored at -80°C until analysis. This study was approved by the Ethics Committee of Beijing Hospital (2016BJYYEC-121-02) and the written informed consent was obtained from each patient.

### Coronary angiography

Coronary angiographies were performed by experienced cardiologists using standard techniques in all study patients. All targeted coronary lesions of the patients were analyzed by the built-in QCA software of the Allura Xper FD20 Angiography System (Philips Healthcare, Netherlands). According to the classification of the American Heart Association Grading Committee, coronary arteries were divided into 15 segments. Coronary artery segments were carefully selected by cardiologists on the basis of smooth luminal borders and the absence of stenosis. All of the coronary arteries were injected and at least two views of the right coronary arteries and four views of the left coronary arteries were evaluated. The existence of CAD was analyzed by two experienced interventional cardiologists and was defined as the presence of one or more coronary arteries with 50% or more stenosis in the main epicardial coronary arteries. In the event of a disagreement, the opinion of a third observer was required, and the final decision was made by consensus of all three observers. To test the extent and severity of ischemia caused by the lesion, the Gensini scores (15, 16) were calculated in 2047 patients who did not have previous percutaneous artery interventions (PCI). Each lesion was assigned a score according to the percentage of stenosis, and multiplied by the coefficient defined for each major coronary artery and segment. The Gensini score of each patient was obtained by summing up the results. Subjects with Gensini scores of the top tertiles were referred as severe CAD.

### Measurement of 1,5-AG and other variables

Serum 1,5-AG was measured by KingMed Diagnostics using Pyranose oxidase assay kit from Beijing Strong Biotechnologies, Inc. on a Roche Modular 702 system. Serum samples were thawed and mixed at room temperature. 1,5-AG concentration was measured according to the manufacturer’s instructions. FBG, HbA1c, total cholesterol (TC), triglycerides (TG), high-density lipoprotein cholesterol (HDL-C), low-density lipoprotein cholesterol (LDL-C), creatinine (CREA), uric acid (UA) and estimated glomerular filtration rate (eGFR) were measured at the clinical laboratory of Beijing Hospital by using assay kits from Sekisui Medical Technologies (Osaka, Japan) on a Hitachi 7180 chemistry analyzer. Two quality control materials, prepared by mixed fresh serum samples, were analyzed with patient samples in each run in 1,5-AG assays to monitor the performance of the measurements. The average inter- assay CV was below 2%. Previous studies have shown this 1,5-AG assay to be highly reliable even in long-term stored samples (17). In the 2945 subjects measured, 100 samples had 1,5-AG concentration below the limit of detection, on this occasion, the lowest measured concentration was inputted.

### Statistical analysis

Data were presented as percentages or medians and interquartile ranges (IQR). Baseline characteristics of the study population were compared across categories of 1,5-AG (≥14 µg/mL, <14µg/mL) in subjects with and without diagnosed DM. We used the Chi-squared test or Fisher exact test for categorical variables and the Kruskal-Wallis test for continuous variables. Correlations between 1,5-AG and conventional CAD risk factors were analyzed by Spearman nonparametric test.

To analyze the associations of 1,5-AG with the incidence of CAD and CAD severity defined by Gensini scores, multivariable logistic regression or linear regression analysis were performed. We divided the subjects with and without diabetes into two groups based on a cut point of 14 μg/mL. Those without diagnosed diabetes and 1,5-AG≥14 μg/mL served as the common reference group. Odds ratios (ORs) for CAD versus non-CAD, high versus low Gensini scores, or regression coefficients β for Gensini score, and the corresponding 95% confidence intervals (CIs) were evaluated. Potential confounding variables, including age, gender, current smoking, obesity or overweight, hypertension, dyslipidemia, stroke, family history of premature CAD, statin use, and FBG or HbA_1C,_ were controlled in the regression models.

We used restricted cubic splines with five knots located at the 5th, 27.5th, 50th, 72.5th, and 95th percentiles and centered at the 14 μg/mL to explore whether there is a non-linear relationship between 1,5-AG and incident CAD and severity. Besides, two-piecewise linear regression model was also used to further explain the non-linearity and examine the threshold effect of the 1,5-AG on CAD and severity using a smoothing function. In the model, the threshold level (i.e.,turning point) was determined using trial and error, including selection of turning points along a pre-defined interval and then choosing the turning point that gave the maximum model likelihood. We also conducted a log likelihood ratio test to examine the statistical significance.

In addition, the subgroup analyses and interaction tests were performed using stratified binary logistic regression model or linear regression. In order to further examine the robustness of associations between 1,5-AG and incident CAD and severity, we performed several sensitivity analyses: additionally adjusting for the serum concentrations of other biochemical parameters, including TC, TG, HDL-C, LDL-C, UA, Crea and eGFR; excluding participants with 1,5-AG below the limit of detection; using 1,5-AG concentration as a continuous independent variable instead of the category variable with a cutoff for 1,5-AG of ≥ 14 μg/mL, respectively.

The analyses were performed using SPSS 26.0 (Windows SPSS, Inc.) and R packages (http://www.r-project.org). All reported *P* values were two-tailed, *P* value<0.05 was considered significant.

## Results

### Characteristics of the study participants

At baseline, participants were a mean of 65 years old, 62.4% males and 49.6% had a diagnosed DM. The participants were classified into two groups of undiagnosed and diagnosed DM, the baseline characteristics by 1,5-AG categories (≥14 μg/mL, <14 μg/mL) within the two groups and among four subgroups were compared. As shown in **Table 1**, in patients with diagnosed DM (n=1462), 57.7% had 1,5-AG concentration <14mg/mL, while in patients without DM (n=1483), only 7.8% had low 1,5-AG levels (<14 μg/mL). In both diagnosed and undiagnosed DM, subjects with 1,5-AG <14 μg/mL had significantly higher FBG and HbA1C levels compared to those with 1,5-AG ≥14 mg/mL. The percent of coronary angiography defined CAD, as well as Gensini scores, were also higher in low 1,5-AG (< 14 μg/mL) subjects compared to those with higher 1,5-AG (≥ 14 μg/mL). In addition, age, BMI, SBP, percent of BMI ≥ 24 kg/m^2^, HTN, DM, dyslipidemia, and statin use all significantly increased among subgroups (*P* values for trend <0.05). FBG, HbA1C, coronary angiography defined CAD, and Gensini scores were also significantly increased from undiagnosed DM to diagnosed DM and across different 1,5-AG categories (*P* values for trend <0.05).

**Table 1:** Characteristics of the study participants by categories of 1,5-anhydroglucitol (1,5-AG) at baseline in subjects with and without diagnosed diabetes.

		No diagnosed diabetes, n =1483		Diagnosed diabetes, n =1462	
	Total	1,5-AG ≥ 14 μg/mL	1,5-AG < 14 μg/mL	1,5-AG ≥ 14 μg/mL	1,5-AG < 14 μg/mL
N	2945	1367	116	619	843
*1,5-AG, μg/mL	20.8 (10.1, 30.2)	28.1 (21.8, 35.2)	10.1 (7.5, 12.2) #	23.5 (18.5, 30.5)	5.9 (3.5, 9.4) #
1,5-AG, μg/mL, range	1.0, 99.3	14.0, 99.3	1.0, 13.99	14.0, 80.6	1.0, 13.99
*FBG, mmol/L	5.9 (5.1, 7.5)	5.2 (4.9, 5.8)	5.5 (5.0, 6.1) #	7.4 (6.0, 8.6)	7.9 (6.4, 10.0) #
*HbA1C, %	6.2 (5.8, 7.1)	5.8 (5.5, 6.0)	6.0 (5.6, 6.2) #	6.3 (5.9, 6.8)	7.6 (6.8, 8.6) #
*Age, y	65.0 (58.0, 74.0)	64.0 (57.0, 73.0)	69.0 (61.0, 77.8) #	67.0 (60.0, 74.0)	66.0 (58.0, 76.0)
Male, %	62.4	62.9	59.5	64.5	60.7
*BMI, kg/m^2^	25.4 (23.4, 27.7)	25.1, (23.2, 27.3)	25.7 (23.8, 28.7) #	25.8 (23.9, 27.9)	25.5 (23.5, 28.0)
*BMI ≥ 24 kg/m^2^, %	67.9	65.2	73.7	74.1	70.0
*SBP, mmHg	135.0 (123.0, 149.0)	133.0 (121.0, 146.0)	134.5 (123.0, 150.8)	136.0 (124.0, 150.0)	139.0 (127.0, 151.0) #
DBP, mmHg	79.0 (70.0, 85.0)	79.0 (71.0, 85.0)	79.5 (70.3, 86.8)	78.0 (70.0, 85.0)	78.0 (70.0, 85.0)
*TC, mmol/L	3.8 (3.2, 4.5)	3.9 (3.3, 4.5)	3.7 (3.2, 4.6)	3.8 (3.2, 4.5)	3.7 (3.2, 4.4)
*TG, mmol/L	1.3 (0.9, 1.8)	1.2 (0.9, 1.7)	1.2 (0.9, 1.7)	1.3 (1.0, 1.8)	1.3 (1.0, 1.9)
*HDL-C, mmol/L	1.0 (0.9, 1.2)	1.1 (0.9, 1.3)	1.0 (0.8, 1.2)	1.0 (0.9, 1.2)	1.0 (0.9, 1.1) #
LDL-C, mmol/L	2.3 (1.8, 2.8)	2.3 (1.8, 2.9)	2.3 (1.8, 2.9)	2.3 (1.8, 2.9)	2.2 (1.7, 2.8)
Crea, μmol/L	70.0 (60.0, 81.0)	70.0 (60.0, 80.0)	72.0 (62.0, 81.0)	71.0 (60.0, 83.0)	69.0 (57.0, 80.0) #
*UA, μmol/L	320.0 (266.0, 379.0)	327.0 (270.0, 390.0)	305.0 (251.5, 382.5)	333.0 (280.0, 384.0)	300.5 (254.0, 354.0) #
eGFR, mL/min per 1.73 m^2^	95.5 (81.6, 110.3)	95.8 (83.1, 109.2)	91.9 (77.9, 104.7) #	93.3 (78.7, 109.1)	97.6 (80.3, 114.4) #
Current smoker, %	31.8	32.1	32.5	30.4	32.7
*Hypertension, %	60.7	66.4	73.5	73.8	74.3
*Diabetes, %	49.6	0	0	100	100
*Dyslipidemia, %	44.1	41.5	38.8	45.5	48.7
History of stroke, %	10.7	10.2	10.3	9.9	12.0
Family history of premature CAD, %	7.7	8.2	4.5	8.6	7.3
*Take statins continuously over 1 year, %	25.3	22.4	19.0 #	27.6	29.2
*Coronary angiography defined CAD, %	78.6	70.2	77.6	83.8	88.5 #
*Gensini score	12.0 (2.0, 37.5)	6.0 (1.0, 24.5)	9.5 (2.8, 29.5) #	18.3 (4.0, 45.5)	24.0 (6.0, 53.0) #

CAD, coronary artery disease; BMI, body mass index; SBP, Systolic blood pressure; DBP, Diastolic blood pressure; FBG, fasting blood glucose; TC-total cholesterol; TG- triglyceride; HDL-C, high density lipoprotein cholesterol; LDL-C, low density lipoprotein cholesterol; Crea, creatinine; UA, uric acid; eGFR, estimated glomerular filtration rate

Data are median (interquartile range) for continuous variables, or percentage for categorical variables. * P values for trend <0.05 among four groups. Significant differences (# P<0.05) compared with groups of 1,5-AG ≥ 14 μg/mL in no diagnosis of diabetes or diagnosed diabetes, respectively

### Correlations between 1,5-AG and conventional CAD risk factors

Spearman correlations between 1,5-AG and other CAD risk factors were presented in **Table 2**. In subjects without a diagnosis of DM, serum 1,5-AG levels were significantly negatively correlated with age, BMI (*P<*0.001) and SBP (*P<*0.05), and positively correlated with UA (*P<*0.001) and eGFR (*P<*0.05). In subjects with a diagnosed DM, 1,5-AG was found to be negatively correlated with SBP (*P<*0.01) and eGFR (*P<*0.001), and positively correlated with TC (*P<*0.05), HDL-C, Crea and UA (*P<*0.001). In all the patients including both with and without DM, serum 1,5-AG was significantly negatively associated with age, BMI, SBP, and TG (*P*<0.001), and positively associated with TC (*P<*0.01) and HDL-C (*P<*0.001) levels. In addition, serum 1,5-AG was significantly negatively associated with Gensini scores in all subjects (*P<*0.001) and subjects with DM (*P<*0.01), but no association was found in subjects without DM. The weak associations found between 1,5-AG and TC were probably caused by the use of statins.

**Table 2:** Spearman correlation (r) of 1,5-AG with other clinical parameters.

	All	No diagnosed diabetes	Diagnosed diabetes
Age	-0.061***	-0.093***	0.055*
FBG	-0.404***	-0.084**	-0.179***
HbA1C	-0.672***	-0.196***	-0.686***
BMI	-0.072***	-0.089***	0.004
SBP	-0.110***	-0.062*	-0.076**
DBP	0.006	-0.026	-0.010
TC	0.052**	-0.016	0.055*
TG	-0.067***	-0.015	-0.032
HDL-C	0.138***	-0.008	0.130***
LDL-C	0.037	-0.006	0.034
Crea	0.044*	0.006	0.108***
UA	0.158***	0.103***	0.226***
eGFR	-0.024	0.053*	-0.120***
Gensini score	-0.185***	-0.015	-0.093**

*p< 0.05, **p< 0.01, ***p< 0.001

### Multivariable analysis between categories of 1,5-AG with CAD, gensini scores and severe CAD

The associations between 1,5-AG and the presence of CAD, the Gensini scores, as well as CAD severity (high vs low Gensini score), were analyzed by the multivariable analysis in subjects with and without diagnosed DM, with those no diagnosed DM and 1,5-AG ≥14 µg/mL served as the common reference group. As shown in **Table 3**, the associations significantly increased, in a dose-response manner, from undiagnosed DM with 1,5-AG ≥ 14µg/mL and < 14µg/mL to DM with 1,5-AG ≥ 14µg/mL and < 14µg/mL in order (*P* values for trend <0.01). The highest ORs for the prevalence of CAD, elevated Gensini scores and severe CAD were found in subjects with diagnosed DM and 1,5-AG < 14 µg/mL after adjusting for age, gender, and other CAD risk factors (Model 1& 2). The associations were attenuated but remained significant after adjustment for FBG (Model 3) and HbA_1C_ (Model 4).

**Table 3:** Adjusted β/OR (95% CI) and p-value of baseline diabetes-specific categories of 1,5-AG with prevalent coronary angiography defined CAD, Gensini score and severe CAD.

	Model 1	Model 2	Model 3	Model 4
Prevalent coronary angiography defined CAD				
**No diagnosed diabetes**				
1,5-AG ≥ 14 μg/mL	1.00 (reference)	1.00 (reference)	1.00 (reference)	1.00 (reference)
1,5-AG < 14 μg/mL	1.377 (0.864, 2.195), 0.178	1.597 (0.963, 2.648), 0.070	1.441 (0.861, 2.410), 0.164	1.130 (0.595, 2.146), 0.708
**Diagnosed diabetes**				
1,5-AG ≥ 14 μg/mL	2.087 (1.625, 2.681), <0.001	1.993 (1.539, 2.581), <0.001	1.271 (0.942, 1.714), 0.117	1.603 (1.164, 2.206), 0.004
1,5-AG < 14 μg/mL	*3.318 (2.591, 4.249), <0.001	*3.192 (2.467, 4.131), <0.001	*1.766 (1.284, 2.430), <0.001	1.641 (1.103, 2.440), 0.014
*P* for trend	<0.001	<0.001	<0.001	0.002
**Gensini score**				
**No diagnosed diabetes**				
1,5-AG ≥ 14 μg/mL	0 (reference)	0 (reference)	0 (reference)	0 (reference)
1,5-AG < 14 μg/mL	3.508 (-3.729, 10.746) 0.342	4.382 (-2.946, 11.709), 0.241	3.737 (-3.614, 11.087), 0.319	-1.077 (-10.696, 8.543), 0.826
**Diagnosed diabetes**				
1,5-AG ≥ 14 μg/mL	10.906 (7.244, 14.568), <0.001	10.848 (7.141, 14.554), <0.001	5.322 (1.132, 9.511), 0.013	9.069 (4.442, 13.696), <0.001
1,5-AG < 14 μg/mL	*18.008 (14.640, 21.377), <0.001	*17.476 (14.060, 20.893), <0.001	9.350 (4.981, 13.719), <0.001	9.700 (4.181, 15.220), <0.001
*P* for trend	<0.001	<0.001	<0.001	<0.001
**Prevalent severe CAD**				
**No diagnosed diabetes**				
1,5-AG ≥ 14 μg/mL	1.00 (reference)	1.00 (reference)	1.00 (reference)	1.00 (reference)
1,5-AG < 14 μg/mL	1.771 (0.974, 3.223), 0.061	*1.974 (1.044, 3.734) 0.036	1.681 (0.877, 3.225), 0.118	1.388 (0.603, 3.196), 0.441
**Diagnosed diabetes**				
1,5-AG ≥ 14 μg/mL	2.756 (2.018, 3.765), <0.001	2.751 (1.979, 3.825), <0.001	1.313 (0.885, 1.948), 0.176	2.308 (1.528, 3.484), <0.001
1,5-AG < 14 μg/mL	*5.060 (3.760, 6.811), <0.001	*5.164 (3.777, 7.059), <0.001	*1.933 (1.285, 2.908), 0.002	2.555 (1.569, 4.161), <0.001
*P* for trend	<0.001	<0.001	0.002	<0.001

ORs, odds ratios; CI, Confidence Intervals; CAD, coronary artery disease; SD, standard deviation

Model 1: age and gender. Model 2: variables in model 2 plus current smoker, obesity or overweight, hypertension, dyslipidemia, stroke, family history of premature CAD and statin use. Model 3: variables in model 2 plus FBG (mmol/L). Model4: variables in model 2 plus HbA_1C_ (%). P values for overall trend were calculated by modeling the category medians as a continuous variable. Significant differences (* P<0.05) between 1,5-AG categories within diabetes group (no diagnosis of diabetes or diagnosed diabetes)

**Figure 1:**
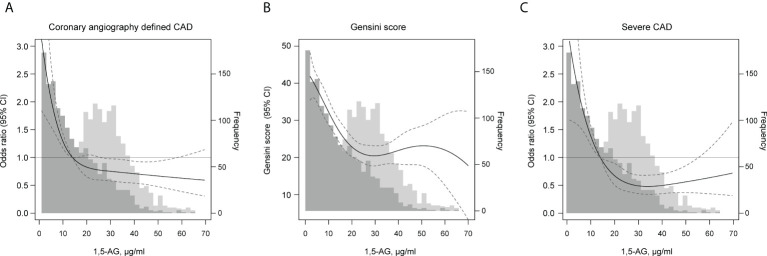
Adjusted associations of serum 1,5-AG levels with **(A)** coronary angiography defined CAD, **(B)** Gensini scores and **(C)** severe CAD in the BHAS study, N = 2945. 1,5-AG was modeled using restricted cubic splines (solid line) with knots at the 5th, 27.5th, 50th, 72.5th, and 95th percentiles. The concentration of 1,5-AG of 14 μg/mL was used as the reference point for **(A)** and **(C)**. 95% CIs are shown with the dashed lines. Models are adjusted for age, gender, current smoker, obesity or overweight, diabetes, hypertension, dyslipidemia, stroke and family history of premature CAD. Frequency histograms are shown for persons without diabetes (light gray bars) and for persons with diabetes (dark gray bars).

In subjects with diagnosed DM, compared with 1,5-AG ≥ 14 μg/mL, subjects with 1,5-AG < 14 μg/mL had more significant associations with the prevalent CAD, elevated Gensini scores and severe CAD in Model 1 &2 (*P*<0.05). The association remained significant after additional adjustment for FBG (Model 3) but not HbA1c (Model 4). However, in subjects without DM, compared with 1,5-AG ≥ 14 μg/mL, 1,5-AG < 14 μg/mL had no associations with the prevalence of CAD and elevated Gensini scores. Weak associations were found between 1,5-AG < 14 μg/mL and severe CAD in Model 2. The associations were no longer significant after further adjusting for FBG (Model 3) or HbA_1C_ (Model 4). These results indicated that, in the categorical analyses, the associations of low 1,5-AG (< 14 μg/mL) with the prevalent CAD, elevated Gensini scores and CAD severity were largely confined to persons with diagnosed DM. In sensitivity analyses, associations of 1,5-AG with CAD, elevated Gensini scores and severe CAD remained essentially unchanged after further adjustment for other biochemical parameters, including TC, TG, HDL-C, LDL-C, UA, Crea and eGFR (**Supplementary Table 1**). Similar associations were observed when restricting the analyses to participants with 1,5-AG levels above the limit of detection (n=2845) (**Supplementary Table 1**) and using 1,5-AG concentration as a continuous independent variable instead of the category variable with a cutoff of 1,5-AG ≥ 14 μg/mL. (**Figure 1**, **Supplementary Figure 1**).

### Analysis of nonlinear relationship between 1,5-AG and incident CAD and severity

The restricted cubic splines were used to flexibly model and visualize the distribution of 1,5-AG and its relation with CAD, elevated Gensini scores and CAD severity. As shown in the histograms (**Figure 1**), the distributions of 1,5-AG was substantially different in subjects with and without a diagnosis of DM. In subjects without DM, the distribution of 1,5-AG is roughly normal (light gray bars), however, in persons with diagnosed DM, the distribution of 1,5-AG was non-normal and highly right skewed (black bars). When modeled with restricted cubic splines, the lowest level of 1,5-AG tended to be associated with the highest risk of incident CAD, elevated Gensini scores and severe CAD when adjusted for age, gender, current smoker, obesity or overweight, diabetes, hypertension, dyslipidemia, stroke, family history of premature CAD and statin use. The association was not linear across the entire distribution, we observed a threshold effect, with apparent dose-response associations at lower levels but little evidence of associations at higher levels. In **Table 4**, by using the two-piece wise linear regression model, the turning point of 1,5-AG was calculated as 14.0 μg/mL for prevalent CAD. Therefore, on the left of the turning point, the adjusted ORs (95%CI) were 0.420 (0.263, 0.670) (*P <*0.001), indicating significant associations with CAD. However, on the right of the turning point, the adjusted associations were not significant. Compared with the linear model, the differences found were statistically significant (*P* for log likelihood ratio tests were <0.001). Interestingly, the turning points for the Gensini scores and severe CAD were 29.7 μg/mL and 28.8 μg/mL respectively, which were much higher than that of CAD.

**Table 4:** Threshold effect analysis of 1,5-AG (per 1-SD increment) on coronary angiography defined CAD, Gensini score and the severity of CAD.

	Crude β/OR (95% CI) *p*-value	*Adjusted β/OR (95% CI)*p*-value
**CAD**		
1,5-AG < 14.0 μg/mL	0.296 (0.197, 0.445), <0.001	0.420 (0.263, 0.670), <0.001
1,5-AG ≥14.0 μg/mL	0.875 (0.776, 0.986), 0.029	0.969 (0.847, 1.108), 0.644
*P* for log likelihood ratio test	<0.001	0.002
**Gensini score**		
1,5-AG < 29.7 μg/mL	-9.018 (-11.240, -6.796), <0.001	-4.674 (-7.189, -2.159), <0.001
1,5-AG ≥ 29.7 μg/mL	2.189 (-1.335, 5.713) 0.2235	1.963 (-1.415, 5.340), 0.255
*P* for log likelihood ratio test	<0.001	0.008
**Severe CAD**		
1,5-AG < 28.8 μg/mL	0.433 (0.360, 0.520), <0.001	0.589 (0.464, 0.746), <0.001
1,5-AG ≥ 28.8 μg/mL	1.224 (0.962, 1.558), 0.100	1.221 (0.935, 1.595), 0.143
*P* for log likelihood ratio test	<0.001	<0.001

Crude: no adjustment

*Adjusted for age, gender, current smoker, obesity or overweight, diabetes, hypertension, dyslipidemia, stroke and family history of premature CAD

### Stratified analysis of the associations between 1,5-AG with CAD, gensini scores and CAD severity

In order to examine the robust associations of 1,5-AG with CAD, associations between 1,5-AG levels and the incidence of CAD, the Gensini scores and CAD severity were respectively analyzed in different subgroups, such as different age, gender, BMI (<24kg/m^2^, ≥24kg/m^2^), smoking status, hypertension, dyslipidemia, and with or without diagnosed DM, stroke, and family history of premature CAD. Interactions between 1,5-AG and these factors on the distribution of CAD, Gensini scores and severe CAD were also analyzed. As shown in **Supplementary Figure 1**, the results suggested that when 1,5-AG was used as continuous variables, each SD increase was associated with 15% decrease of prevalent CAD, 2 point lower of Gensini scores and 18% decrease of severe CAD. The associations were apparently more significant in persons with diagnosed DM compared with non-DM. These results were similar with those when 1,5-AG was modeled as a categorical variable (**Table 3**). However, only the interaction by DM for Gensini score was observed (*P* for interaction =0.029, **Supplementary Figure 1B**). Although the ORs (95% CI) for CAD and severe CAD were different in DM and non-DM patients [0.771 (0.650-0.914) vs 0.919 (0.799-1.057) for CAD, and 0.716 (0.580-0.884) vs 0.909 (0.751-1.100) for severe CAD], no significant effect modification by DM was found (*P* for interaction > 0.05). We did not observe interactions by age, sex, BMI, current smoker, dyslipidemia, stroke and family history of premature CAD for the presence of CAD, Gensini score and CAD severity (all *P* for interaction > 0.05).

## Discussion

In the present study, we analyzed the associations between serum 1,5-AG with the prevalence of CAD and CAD severity as assessed by the Gensini score system in Chinese patients underwent coronary angiography. The results showed that low concentrations of 1,5-AG were independently associated with the prevalence of CAD, elevated Gensini scores and severe CAD after adjusting for age, gender and other CAD risk factors. More significant associations remain in DM patients after further adjusting for FBG or HbA_1C_. Nonlinear relationship and threshold effects of 1,5-AG were found for prevalent CAD, elevated Gensini scores, and CAD burden, with the 1,5-AG turning points of 14.0μg/mL, 29.7μg/mL, and 28.8 μg/mL respectively.

DM is a disorder of impaired glucose homeostasis and is one of the important risk factors for cardiovascular diseases, including CAD and related mortality. Recent epidemiological, clinical and experimental data have suggested that effective controlling of blood glucose in the nonfasting state, especially the postprandial period, can reduce the risk of macroangiopathic complications of diabetes (7, 8, 18). Evaluating glycemic control is currently based on the self-monitoring of blood glucose and clinical testing for HbA1c, which is a surrogate biochemical marker of the average glycemia level over the previous 2-3 months (4). Although HbA1c provides a valuable, standardized and evidence-based parameter that is relevant for clinical decision making, there are circumstances that HbA1c levels are insufficient to predict the risk of adverse outcomes (19–21). Moreover, it was well established previously that although postprandial glycaemia can influence HbA1c concentration, glycated haemoglobin is not sensitive for short-lasting, transient hyperglycaemia (22). 1,5-AG is an emerging glycometabolic marker that complements HbA1c in terms of short-term glycemic control, postprandial hyperglycemia and glucose fluctuation (9, 10, 12–14). Serum 1,5-AG concentration is found to be relatively stable in normalglycemia state and rapidly falls reflecting not only chronic, but also short-lasting hyperglycaemic episodes. Therefore, measurement of 1,5-AG is important to identify transient hyperglycaemia and related CAD risks in DM patients and non-DM individuals.

Previous studies have reported that 1,5-AG levels are associated with vascular endothelial dysfunction (23), oxidative stress (24), carotid atherosclerosis (25, 26) and the prevalence of CAD (9, 27). In one previous prospective study of approximately 2000 persons in Japan, 1,5-AG at baseline was significantly associated with incident cardiovascular events during 11 years of follow-up (28). In the ARIC study, a community-based prospective cohort study, low 1,5-AG was associated with subclinical cardiovascular disease, cardiovascular outcomes and mortality in persons with a history of DM (14). However, the relationship of 1,5-AG with CAD or CAD events were not consistent in the population without diagnosis of DM (14, 29–31). The associations between 1,5AG and CAD were also studied in patients undergoing coronary angiography (32, 33), and found that the 1,5-AG levels were independently associated with CAD, and serum 1,5-AG value predicts CAD related adverse events even in non-DM patients without CAD. However, these studies had a relatively strict exclusion criteria and small number of participates which may have limited representations. In addition, whether the association between 1,5-AG and CAD is modified by DM is not clear. In the current study, the relationship of 1,5-AG with the prevalent CAD, Gensini scores, and CAD severity were analyzed in Chinese subjects undergoing coronary angiography in both DM and non-DM patients.

We first analyzed the baseline characteristics in diagnosed DM and non-DM by low (<14 μg/mL) and high (≥14 μg/mL) 1,5-AG categories. In both diagnosed and undiagnosed DM, lower 1,5-AG was associated with not only significantly higher FBG and HbA1C levels, but also higher Gensini scores. These results were in accordance with the previous reports (34, 35). Multivariable analysis showed that the associations robustly dose-reponse increased from undiagnosed DM with 1,5-AG ≥ 14µg/mL to DM with 1,5-AG < 14µg/mL even after adjusting for FBG or HbA_1c._ The associations of low concentrations of 1,5-AG with CAD, elevated Gensini score and severe CAD were more significant in persons with diagnosed DM. However, only significant modification effect of DM on the relationship of 1,5-AG with elevated Gensini score was found. These results suggested that measurement of serum 1,5-AG value may be useful for not only evaluation of glucose control but also CAD risk assessment in high risk populations, especially in DM patients.

Different values have been reported for blood 1,5-AG concentrations depending on the racial/ethnic groups, being higher in Asians and Africans compared to Caucasians (36, 37). In the previous study, 1,5-AG concentration of 10.0 μg/mL (14, 29, 32, 38) and 14.0 μg/mL (28, 31, 39) have been used as the cut-off values for defining exposure to hyperglycemia, potentially representing a subset of the population with higher post-prandial glycemic peaks. In our study, 57.7% of the subjects with diagnosed DM had 1,5-AG<14.0 μg/mL, and in subjects without DM, 92.2% had 1,5-AG>14.0μg/mL. These results were in accordance with the reports of Dr. Yamanouchi (40), in which 14 μg/mL was recommended as the normal lower limit of 1,5-AG levels. When modeled with restricted cubic splines, our results demonstrated that the lowest level of 1,5-AG was associated with the highest prevalence of CAD, elevated Gensini scores and sever CAD. In addition, the association was not linear across the entire distribution, in which a threshold effect was found. By using the two- piecewise linear regression model, the turning points of 1,5-AG for CAD, high Gensini scores, and severe CAD were calculated. The results suggested that, the linear associations between 1,5-AG and CAD was largely confined in 1,5-AG<14μg/mL. However, the turning points of 1,5-AG for high Gensini scores (29.7 μg/mL) and severe CAD (28.8 μg/mL) were much higher. These results indicate that, for prevention of the occurrence and development of atherosclerosis, more strict control of glycemic fluctuations are needed.

We recognize that our study has some limitations. First, this results are based on a single baseline measurement of 1,5-AG and lack of information on 2-h postprandial glucose. Second, owing to the observational nature of the study, we are also not able to completely rule out the possibility of residual confoundings. Third, since this study was a baseline data of our BHAS, the obtained results require further confirmation in future prospective analysis.

## Conclusion

This study demonstrated that low 1,5-AG levels were independently associated with the prevalence of CAD and the severity of the disease evaluated by Gensini scores, in diagnosed DM patients, even after adjusting for FBG or HbA_1C_. The relationship of 1,5-AG with CAD and severity were not linear with threshold effects existed. Our study added to the evidence regarding the value of 1,5-AG as an effective glycometabolic marker that complements HbA1c in terms of glucose fluctuation and CAD risk assessment in persons with DM. Additional studies are needed to understand its possible utility as a tool for diabetes management in primary and secondary prevention of CAD.

## Data availability statement

The data analyzed in this study is subject to the following licenses/restrictions: The data that support the findings of this study are available from the corresponding author upon reasonable request. Requests to access these datasets should be directed to Jun Dong, jun_dong@263.net.

## Ethics statement

The studies involving human participants were reviewed and approved by The Ethics Committee of Beijing Hospital (2016BJYYEC-121-02). The patients/participants provided their written informed consent to participate in this study.

## Author contributions

JD, WC, XY and FJ were responsible for the study design and execution. WZ, XW, XY finished the coronary angiography procedures and clinical data collection. SW, HM and HL finished all the laboratory measurements and data collection. RY and QZ performed the data analysis. RY and JD wrote the main manuscript. WC edited the manuscript. All authors contributed to the article and approved the submitted version.

## Funding

This work was supported by the CAMS Innovation Fund for Medical Sciences (No. 2021-I2M-1-050), the National Key R&D Program of China (2020YFC2008304), and Beijing Natural Science Foundation (No.7222156).

## Acknowledgments

The authors thank Ms. Mo Wang and Yueming Tang for preparing the patient samples.

## Conflict of interest

The authors declare that the research was conducted in the absence of any commercial or financial relationships that could be construed as a potential conflict of interest.

## Publisher’s note

All claims expressed in this article are solely those of the authors and do not necessarily represent those of their affiliated organizations, or those of the publisher, the editors and the reviewers. Any product that may be evaluated in this article, or claim that may be made by its manufacturer, is not guaranteed or endorsed by the publisher.

## Abbreviations

CAD, coronary artery disease; DM, type2 diabetes mellitus; HTN, hypertension; PCI, percutaneous artery interventions; HbA1c, hemoglobin A1c; FBG, fasting blood glucose; 1,5-AG, 1,5-anhydroglucitol; TC, total cholesterol; TG, triglycerides; HDL-C, high-density lipoprotein cholesterol; LDL-C, low-density lipoprotein cholesterol; CREA, creatinine; UA, uric acid; eGFR, estimated glomerular filtration rate; BMI, body mass index; SBP, systolic blood pressure; DBP, diastolic blood pressure; IQR, interquartile ranges; ORs, Odds ratios; CIs, confidence intervals.
