# Supplementary figures and images for: Nonlinear association of 1,5-anhydroglucitol with the prevalence and severity of coronary artery disease in chinese patients undergoing coronary angiography
Source: Front Endocrinol (Lausanne). 2022 Sep 5;13:978520. doi: 10.3389/fendo.2022.978520 (PMC9483025; doi:10.3389/fendo.2022.978520)

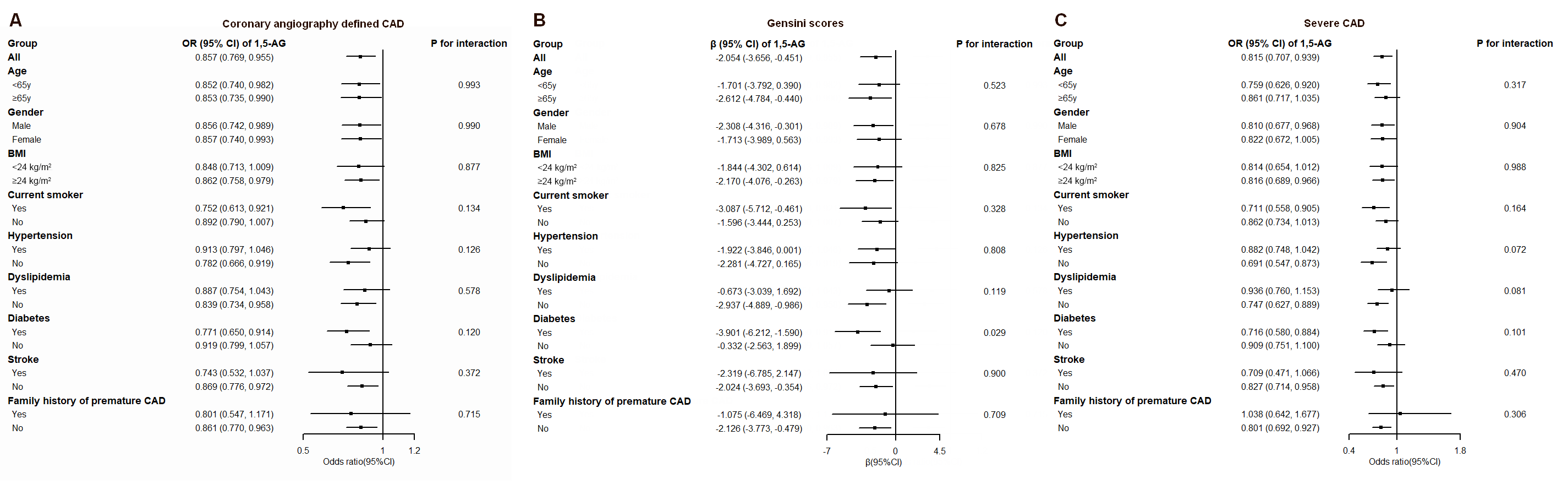

Supplement: Supplementary file 1 [file Image_1.tif]
