# Supplementary material for: Nonlinear association of 1,5-anhydroglucitol with the prevalence and severity of coronary artery disease in chinese patients undergoing coronary angiography
Source: Front Endocrinol (Lausanne). 2022 Sep 5;13:978520. doi: 10.3389/fendo.2022.978520 (PMC9483025; doi:10.3389/fendo.2022.978520)
Supplement: Supplementary file 2 [file Table_1.docx]

**Supplementary Table 1. Associations of 1,5-AG andprevalent coronary angiography defined CAD, Gensini score**

**and severe CAD in basic models and additionally adjusted models**

|  | Model 1 | Model 2 | Model 3 | |
| --- | --- | --- | --- | --- |
| **Prevalent coronary angiography defined CAD** | | | | |
| **No diagnosed diabetes** | | | | |
| 1,5-AG ≥ 14 μg/mL | 1.00 (reference) | 1.00 (reference) | 1.00 (reference) | |
| 1,5-AG < 14 μg/mL | 1.597 (0.963, 2.648), 0.070 | 1.308 (0.749, 2.285), 0.346 | 1.538 (0.926, 2.556), 0.097 | |
| **Diagnosed diabetes** | |  |  | |
| 1,5-AG ≥ 14 μg/mL | 1.993 (1.539, 2.581), <0.001 | 2.087 (1.573, 2.769), <0.001 | 1.998 (1.543, 2.588), <0.001 | |
| 1,5-AG < 14 μg/mL | *3.192 (2.467, 4.131), <0.001 | 2.877 (2.150, 3.850) , <0.001 | *3.107 (2.378, 4.060), <0.001 | |
| *P* for trend | <0.001 | <0.001 | <0.001 | |
| **Gensini score** | | | | |
| **No diagnosed diabetes** | | | | |
| 1,5-AG ≥ 14 μg/mL | 0 (reference) | 0 (reference) | 0 (reference) | |
| 1,5-AG < 14 μg/mL | 4.382 (-2.946, 11.709), 0.241 | 1.709 (-6.464, 9.882), 0.682 | 3.478 (-4.009, 10.965), 0.363 | |
| **Diagnosed diabetes** |  |  |  | |
| 1,5-AG ≥ 14 μg/mL | 10.848 (7.141, 14.554), <0.001 | 10.764 (6.825, 14.702), <0.001 | 10.832 (7.114, 14.550), <0.001 | |
| 1,5-AG < 14 μg/mL | *17.476 (14.060, 20.893), <0.001 | 16.107 (12.270, 19.944), <0.001 | *18.362 (14.795, 21.930), <0.001 | |
| *P* for trend | <0.001 | <0.001 | <0.001 | |
| **Prevalent severe CAD** | | | |  |
| **No diagnosed diabetes** | | | |  |
| 1,5-AG ≥ 14 μg/mL | 1.00 (reference) | 1.00 (reference) | 1.00 (reference) | |
| 1,5-AG < 14 μg/mL | 1.974 (1.044, 3.734), 0.036 | 1.683 (0.792, 3.577), 0.176 | 1.775 (0.927, 3.399), 0.083 | |
| **Diagnosed diabetes** | |  | | |
| 1,5-AG ≥ 14 μg/mL | 2.751 (1.979, 3.825), <0.001 | 3.090 (2.133, 4.476), <0.001 | 2.758 (1.984, 3.834), <0.001 | |
| 1,5-AG < 14 μg/mL | *5.164 (3.777, 7.059), <0.001 | 4.503 (3.144, 6.448), <0.001 | *5.111 (3.705, 7.050), <0.001 | |
| *P* for trend | <0.001 | <0.001 | <0.001 | |

Abbreviations: ORs, odds ratios; CI, Confidence Intervals; CAD, coronary arterydisease; SD, standard deviation.

Basic model (Model 1): Adjusted for age, gender, current smoker, obesity or overweight, hypertension, dyslipidemia, stroke, family history of premature CAD and statin use. Model 2: variables in model 1 plus serum concentrations of other biochemical parameters, including TC, TG, HDL-C, LDL-C, UA, Crea and eGFR. Model 3: variables in model 1 and restricting the analyses to participants without 1,5-AG below the detection limit (n=2845). *P* values for overall trend were calculated by modeling the category medians as a continuous variable. Significant differences (* *P*<0.05) between1,5-AG categories within diabetes groups (no diagnosis of diabetes or diagnosed diabetes).
